# Green synthesis of Zn-doped TIO_2_ nanoparticles from *Zanthoxylum armatum*

**DOI:** 10.1186/s12870-024-05525-3

**Published:** 2024-08-31

**Authors:** Amina Batool, Azizullah Azizullah, Kamran Ullah, Salma Shad, Farman Ullah Khan, Mahmoud F. Seleiman, Tariq Aziz, Umar Zeb

**Affiliations:** 1 Department of Biology, Faculty of Biological and Biomedical Science, The University Haripur, Khyber Pakhtunkhwa, 22620 Pakistan; 2 School of Food and Biological Engineering, Jiangsu University, Zhenjiang, 212013 China; 3 Department of Chemistry, The University of Haripur, Khyber Pakhtunkhwa, 22620 Pakistan; 4 Department of Chemistry, University of Science and Technology Bannu, Khyber Pakhtunkhwa, 28100 Pakistan; 5 Department of Plant Production, College of Food and Agriculture Science, King Saud University, Riyadh, 11451 Saudi Arabia; 6 Faculty of Civil Engineering and Mechanics, Jiangsu University, Zhenjiang, 212013 China

**Keywords:** *Zanthoxylum armatum*, Titanium dioxide, Zinc oxide, Green synthesis, Doping

## Abstract

Green synthesis is an easy, safe, and environmentally beneficial nanoparticle creation method. It is a great challenge to simultaneously improve the capping and stabilizing agent carrier separation efficiency of photocatalysts. Herein, Zn-doped Titanium dioxide (TiO_2_) nanoparticles with high exposure of 360 nm using a UV/visible spectrophotometer were prepared via a one-step hydrothermal decomposition method. A detailed analysis reveals that the electronic structures were modulated by Zn doping; thus, the responsive wavelength was extended to 600 nm, which effectively improved the visible light absorption of TiO_2_. We have optimized the different parameters like concentration, time, and temperature. The peak for TiO_2_ is located at 600 cm-^1^ in FTIR. A scanning electron microscope revealed that TiO_2_ has a definite shape and morphology. The synthesized Zn-doped TiO_2_NPs were applied against various pathogens to study their anti-bacterial potentials. The anti-bacterial activity of Zn-doped TiO_2_ has shown robust against two gram-ve bacteria (*Salmonella* and *Escherichia coli*) and two gram + ve bacteria (*Staphylococcus epidermidis* and *Staphylococcus aureus*). Synthesized Zn-doped TiO_2_ has demonstrated strong antifungal efficacy against a variety of fungi. Moreover, doping TiO_2_ nanoparticles with metal oxide greatly improves their characteristics; as a result, doped metal oxide nanoparticles perform better than doped and un-doped metal oxide nanoparticles. Compared to pure TiO_2_, Zn-doped TiO_2_ nanoparticles exhibit considerable applications including antimicrobial treatment and water purification.

## Introduction

### Green synthesis and its importance

Presently, a growing amount of research is focused on the synthesis of nanoscale metals using chemical, physical, and environmentally friendly synthesis techniques. Green synthesis techniques are gradually replacing physical and chemical techniques due to issues with excessive energy consumption, the production of hazardous substances, the use of complex equipment, and the conditions of synthesis. Aerosol application, UV exposure, and heat breakdown are examples of physical approaches. High temperatures and pressure are needed for each of these techniques. The aerosol technique, for instance, requires a flame temperature of about 2400 K to produce atomized aerosol droplets to produce nanoparticulate metals. Chemical processes always involve toxic and costly reagents, auxiliary dispersion stabilizers, and organic solvents. Green synthesis, on the other hand, uses natural and eco-friendly components. Nanoparticles are small particles with sizes ranging from 1 to 100 nanometers. These materials have gained importance and interest in recent years owing to their large number of applications, because the matter at this scale presents a more compact arrangement of atoms and molecules, generating phenomena and acquiring or enhancing mechanical, optical, catalytic, antifungal, and antibacterial properties that are completely different from those of their macroscopic counterparts. They can be classified based on their composition, shape, and size. The most common types of nanoparticles are metals, metal oxides, and quantum dots. Owing to their unique sizes and properties, nanoparticles have attracted significant attention in various fields including medicine, electronics, energy, and environmental science. By reducing their size, nanoparticles can have a higher surface-to-volume ratio, enabling a greater number of atoms or molecules per volume, which means that less material is needed to obtain the same activity and exhibit other properties.

The synthesis of eco-friendly nanoparticles has garnered significant attention since it is simple, inexpensive to process, can be carried out in natural settings, non-toxic, and is environmentally benign [1, 2]. Particularly exciting is the green synthesis of nanoparticles, which, among other benefits, produces the most stable nanoparticles in a wide variety of shapes and sizes [3]. *Zanthoxylum armatum* is a fragrant, sub-deciduous shrub in the Rutaceae family. It features tiny blooms and alternating impairi pinnate leaves, reaching a maximum length of about 5 m [4]. It is a tiny xerophytic shrub that typically has thorns on its leaflet blades. It can be identified by its small, subglobose, red fruit, dense foliage, prickly trunk and branches, and shrubby form [5, 6]. Plant extracts are becoming an increasingly popular choice to produce nanoparticles due to the features that they possess, which include those of a reducing, coating, and stabilizing agent. In this context, metal oxides that have been produced using plant extract have shown a significant amount of promise in a wide variety of application areas. Specifically, zinc oxide (ZnO) nanoparticles (NPs) produced using plant extract demonstrate non-toxicity, thermal stability, and oxidation resistance [7, 8]. *Zanthoxylum armatum* has a high therapeutic value, long-term sustainability, and its reputation as the “toothache-reliever tree” that captivated the botanical community. Timur (*Zanthoxylum armatum*) commonly referred to as the toothache tree is a significant shrub with significant medicinal, commercial, and ecological value [9, 10]. In addition, it may be used for biological purposes such as monitoring biological activity, transferring genes, serving as a wound dressing material, and possessing antimicrobial and antifungal qualities [11, 12]. According to the research that has been conducted on a variety of plants, Timur, also known as *Zanth-oxylum armatum* DC., is a shrub that belongs to the family Rutaceae. Timur is most prevalent in the northern portion of India because of its proximity to the places where they spend the winter [13, 14]. *Zanthoxylum armatum* has been used as a medication since ancient times to treat a variety of illnesses, including fever, dyspepsia, asthma used to treat bleeding gums, toothaches, and other dental issues [15]. The exquisite oil of Timur contains several phytochemical elements, including lignin, terpenoids, amino acids, flavonoids, flavonol glycosides, fatty acids, alkaloids, and phenolics [16]. A respectable amount of different volatile and aromatic components as well as other many chemicals have been isolated from Timur’s essential oil [17]. Further, due to increasing industrial and agricultural activities, organic pollutants such as organic dyes are increasing tremendously and posing challenges to human health. Numerous organic pollutants are affecting human health; however, Congo Red, which is an azo dye, is a major contributor [18–20]. Its plant components are said to contain extracts with pharmacological properties that include immunomodulatory, anti-tumor, anti-oxidative, anti-inflammatory, and anti-microbial properties [21]. Through nanoscale structures nanotechnology is essential to several significant innovations in the fields of biomedical science, electronics, and optics, [22, 23] mechanics, photo-electrochemical applications, energy science, chemical industry, optoelectronic devices, and catalysis etc. [24, 25]. It was discovered that NPs also showed better photocatalytic dye degradation, which suggests that plant extract provides better functionality and multiple applications to the prepared NPs. Because of this, it is possible that plant extract can pave the way forward for the synthesis of better and more multifunctional NPs [26]. Nanoparticles are of significant interest because of their diverse physical and chemical properties, including optical absorption, melting point, thermal, electrical, and mechanical conductivity [27]. It is focused on the creation of nanoparticles (NPs) and their use in various scientific, medical, industrial, and imaging domains [28]. One of the most prevalent elements in the crust of the earth, titanium is employed extensively because of its special optical, biocompatible, highly reactive, and electrochemical qualities [21, 29]. Titanium dioxide (TiO_2_) is one of the most important pigments. It is widely used because it efficiently scatters visible and absorbs UV light, thereby imparting whiteness, brightness, and opacity when incorporated into a plastic. Titanium dioxide is commercially available in two crystal structures anatase and rutile. It is an insoluble, valuable semiconducting transition metal oxide material having distinct features such as easy to control, low cost, and non-toxicity [30]. TiO_2_ is a fire-resistant, high thermal stability metal oxide that is not categorized as dangerous and it is used in solar cells, chemical sensors, and environmental distillation [31]. Metal oxides are considered the prominent photocatalysis, which improves the photocatalytic activity of doping with metal and non-metal oxides [32]. Furthermore, many researchers have doped many transition metals such as Cr, Mn, Fe, Co, Zn, Cu, and Ni into TiO_2_ metal oxide to change its optical band gap, which helps to extend the absorption range in visible light [33, 34]. Doping of different metal oxides improves their biological activities like anti-microbial activity due to their potential, they have reduced the development of Reactive Oxygen Species (ROS), and it is applied for anti-cancer activities [35].

The United Nations (UN) Sustainable Development Goals (SDGs) are a set of 17 goals adopted in 2015 by 193 countries. The goals are a blueprint for a better world and life for all by 2030, through sustainable economic, environmental, and social development. The SDGs are also known as the Global Goals and are intended to apply equally to all countries. The SDGs are integrated, recognizing that action in one area will affect outcomes in others. Some examples of the goals include No poverty, good health and well-being, Quality education, Gender equality, Decent work, and economic growth, and conserving and sustainably using oceans and their resources. Through sustainable (economic, environmental, and social) development, their overall objective is to create a better world, and a better life for all, by 2030. In short, the 17 SDGs are Goal: No Poverty: End poverty in all its forms everywhere. Goal 2: Zero Hunger: End hunger, achieve food security and improved nutrition, and promote sustainable agriculture. Goal 3: Good Health and Well-being: Ensure healthy lives and promote well-being for all at all ages. The Sustainable Development Goals (SDGs) aim to transform our world. They are a call to action to end poverty and inequality, protect the planet, and ensure that all people enjoy health, justice, and prosperity. They were created with the aim of peace and prosperity for people and the planet while tackling climate change and working to preserve oceans and forests. The SDGs highlight the strong interconnections between the environmental, social, and economic aspects of sustainable development. The Sustainable Development Goals (SDGs), also known as the Global Goals, were adopted by the United Nations in 2015 as a universal call to action to end poverty, protect the planet, and ensure that by 2030 all people enjoy peace and prosperity. The 17 SDGs are integrated to recognize that action in one area will affect outcomes in others, and that development must balance social, economic, and environmental sustainability. Countries have committed to prioritizing progress for those who are furthest behind. The SDGs are designed to end poverty, hunger, AIDS, and discrimination against women and girls. The creativity, know-how, technology, and financial resources from all of society are necessary to achieve the SDGs in every context.

The applications of semiconductor TiO_2_ in the photocatalytic oxidation of many undesirable environmental contaminants and the photoelectrochemical conversion of solar energy have been extensively studied owing to its high photostability, nontoxicity, low cost, and availability. Recently, as documented in the literature, TiO_2_ irradiated by UV light has become the benchmark photocatalyst for degrading environmental contaminants. However, the major drawback of TiO_2_ lies in the fact that the absorbance of only a narrow portion of solar spectra in the UV region is due to the large band gap of TiO_2_. To overcome this limitation, numerous studies have been recently performed. Among these, the photosensitized degradation process has the advantage of harvesting the maximum of solar energy by utilizing visible light for the degradation of the dye-containing wastewaters, which is one of the major environmental contaminations in textile and photographic industries [36].

Nanotechnology has been rapidly developing in the last few decades. Metal oxides, such as TiO_2_, ZnO, SnO_2_, and WO_3_, received significant attention because of their unique catalytic properties and wide range of applications. These oxides are commonly used in catalysts, sensor components, and solar cells. TiO_2_ is a well-known catalytic material with relatively low toxicity and high thermal and chemical stabilities. TiO_2_ has three crystalline structures: rutile, anatase, and brookite. The brookite phase (orthorhombic structure) is of low interest because of the difficulty in synthesizing the pure brookite phase. However, the anatase and rutile phases are extensively exploited in catalytic applications. TiO_2_ has emerged as one of the most promising photocatalysts for the elimination of wastewater pollution due to its excellent properties such as high stability, non-toxicity, low cost, and outstanding photocatalytic activity. However, the inherent electronic structure of TiO_2_ weakens its utilization efficiency of solar energy. More importantly, the short free time and severe surface recombination of photogenerated electrons greatly reduce the efficiency of the photogenerated carriers. Therefore, it is urgent to develop some effective strategies to widen the optical capture range and enhance it [37].

Therefore, it has come to light that both ZnO NPs and the Timur plant (*Zanthoxylum armatum* DC) offer a significant amount of untapped potential within the realm of biological applications. Considering the research presented above, it has been noticed that there are no studies available on their combined effect and the synthesis of ZnO NPs utilizing Timur as a reducing agent. This was found to be the case after reviewing the relevant published research. Because of this, we were inspired to make use of *Zanthoxylum armatum* DC. as a reducing and capping agent and to investigate its possible applications. In this work, we synthesized ZnO NPs by using the plant extract of Timur (*Zanthoxylum armatum* DC) as a reducing agent, a stabilizing agent, and a coating agent. In addition to this, we investigated the synthesized ZnO NPs’ structural and morphological characteristics, as well as nanomaterials were studied for anti-microbial and anti-cancer activity, and various metal oxides were utilized for doping and multiple methods for the synthesis of doped nanoparticles and their biomedical applications. As a result, the purpose of this work is to make modifications to the ZnO NPs so that they may be used in more sophisticated applications.

## Materials and methods

### *Zanthoxylum armatum* leaves collections and processing

**Fig. 1:**
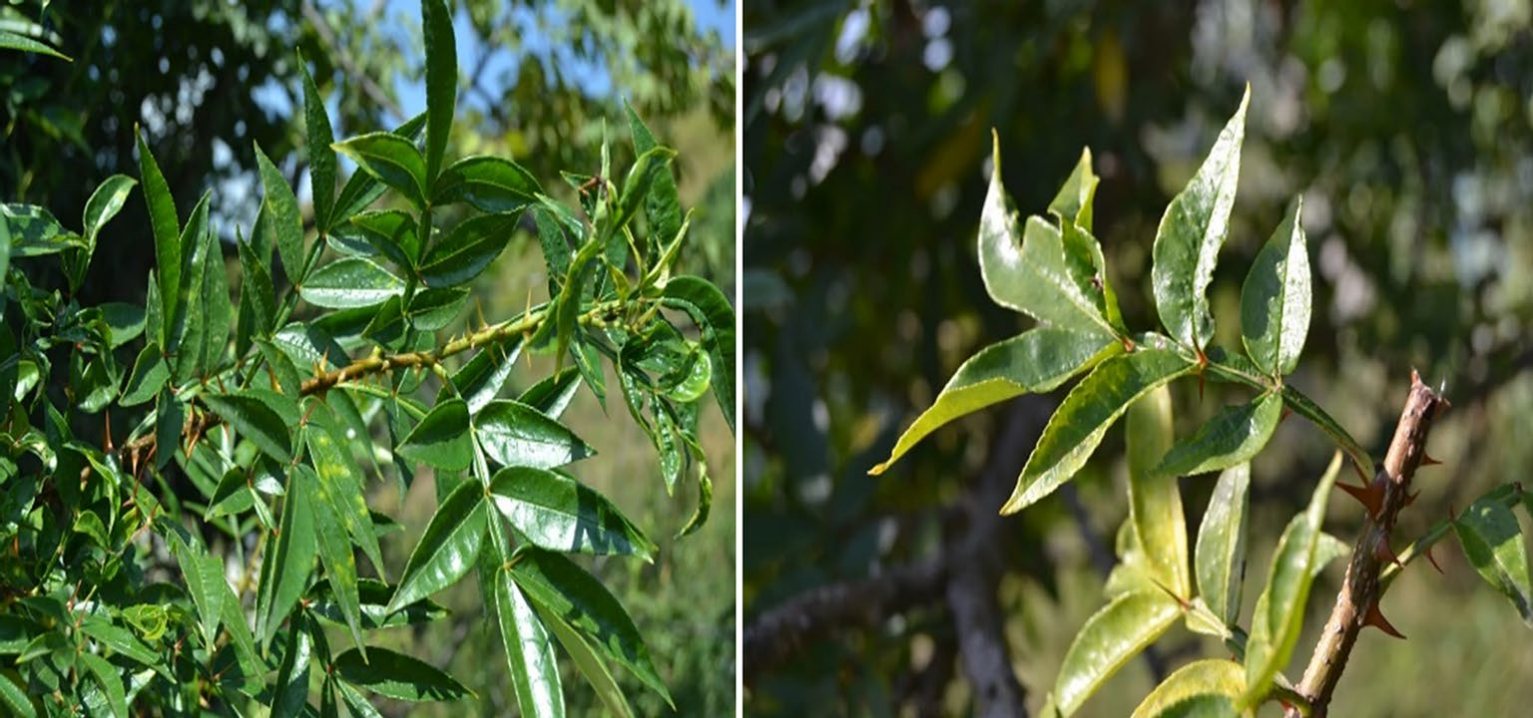
Leaves of *Zanthoxylum armatum*

*Zanthoxylum armatum* leaves as shown in Fig 1 were collected from District Abbottabad and Muzaffarabad Pakistan. The collected leaves were washed with tap water to remove dust and sand particles. The leaves washed were again with deionized water to remove contaminations. After washing, the leaves were dried on paper to remove moisture content. The leaves were cut into small pieces with the help of scissors to prepare the extract [38, 39].

### Preparation of *Zanthoxylum armatum* leaves extract

**Fig. 2:**
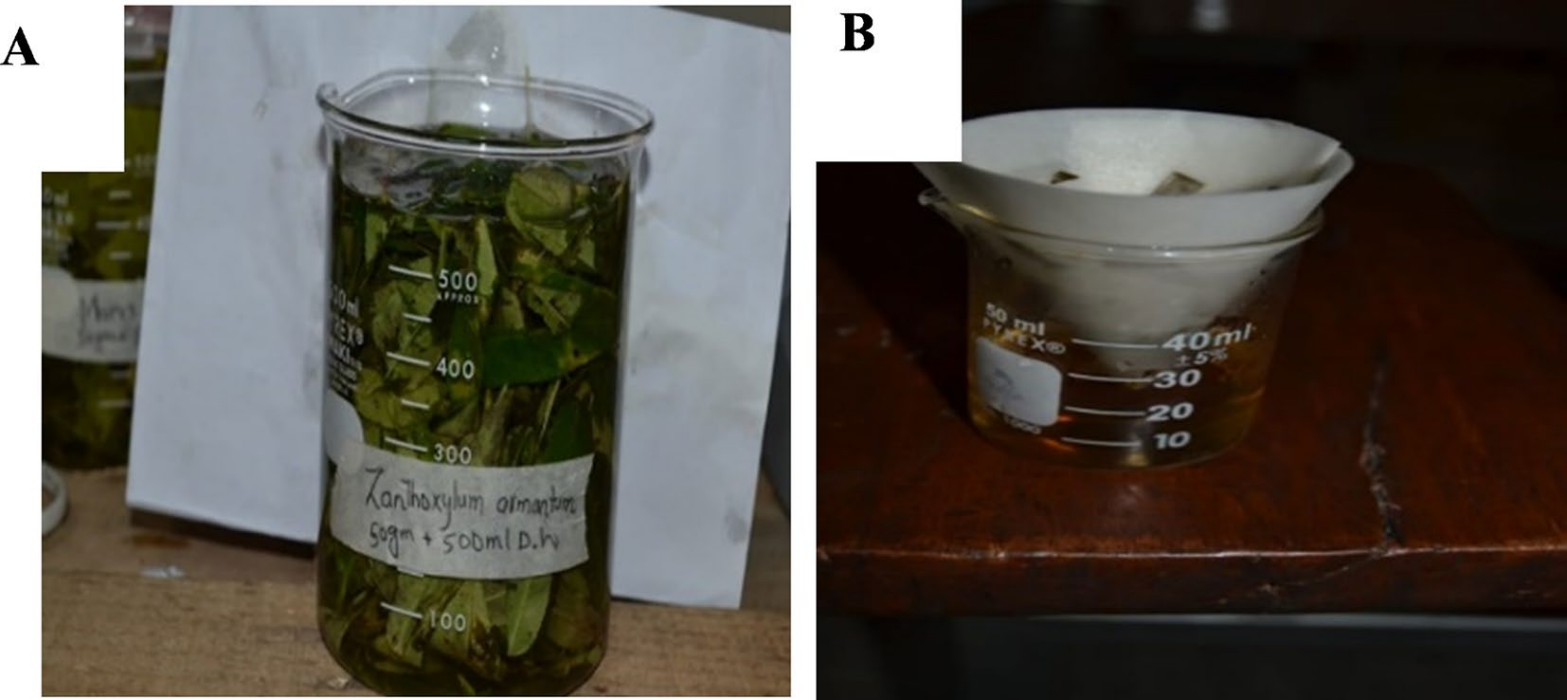
Preparation of *Zanthoxylum armatum* leaves extract

The young leaves of the Timur plant (*Zanthoxylum armatum* DC) 50 g were gathered and dissolved in 500 ml of distilled water. The soxlette contraption was used to extract the substance from these fresh leaves. To manufacture ZnO NPs, a solution of zinc acetate with a concentration of one milligram per milliliter (99.9% purity) was dissolved in 100 milliliters of distilled water and stirred continuously over a hot plate at 80 °C for 60 min. During the agitation of the mixture, 20 ml of plant extract was added, and then the mixture was stirred once more for 30 min. The color of the solution changes to yellow. The temperature of the hot plate stirrer was then raised to 40 °C. After allowing the sediments to settle, they were cleaned many times with distilled water and dried for the whole night at a temperature of 60 °C. The extraction and preparation were filtered out and kept in the refrigerators at 4 °C for 12 h. After drying, the samples were crushed and transported to be characterized as shown in Fig 2.

### Synthesis of TiO2 nanoparticles

**Fig. 3:**
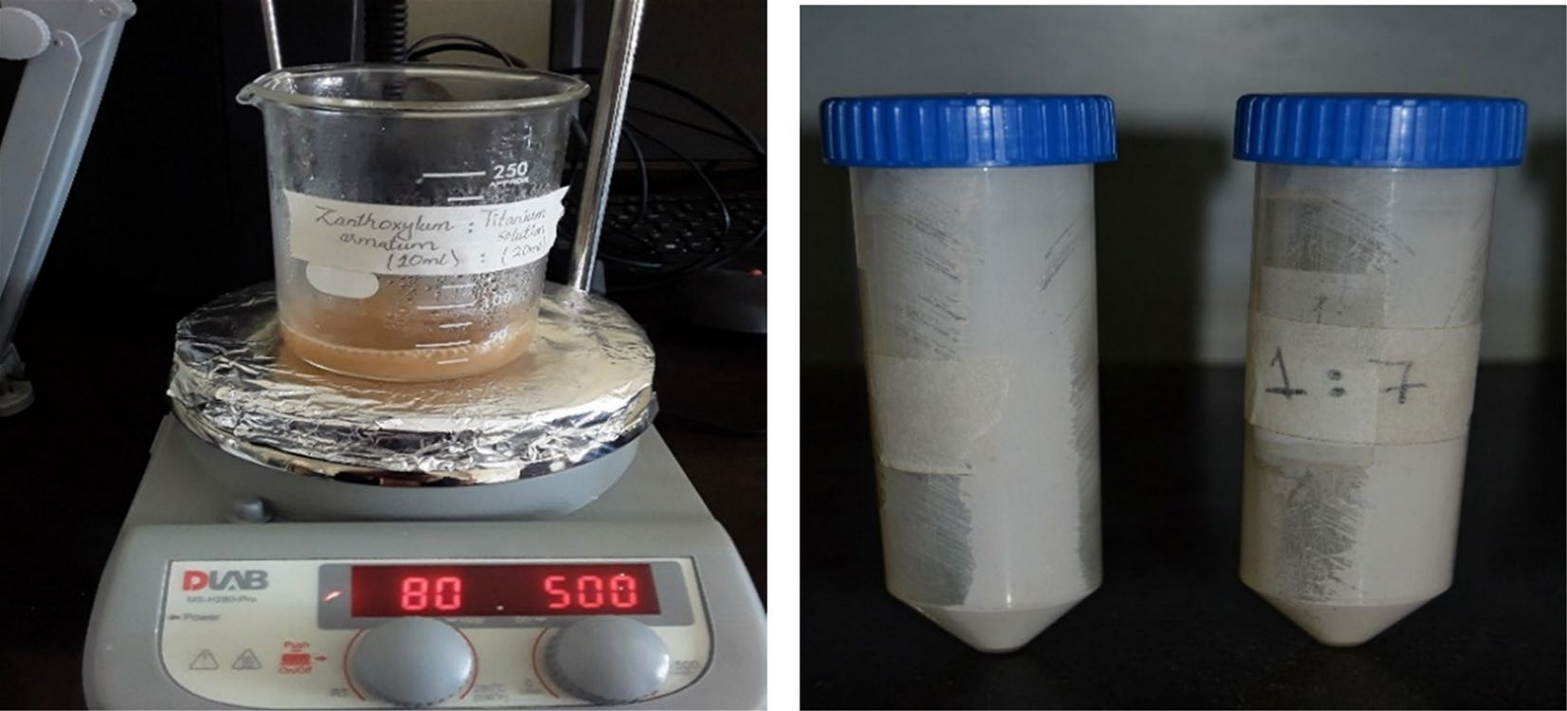
Synthesis of TiO_2_ nanoparticles

Based on the findings from the referred journal, various techniques can be adopted for the preparation of TiO_2_ nanoparticles such as the sol-gel method, instantaneous synthesis method, solvothermal method, microwave-assisted synthesis, simple mixing, and precipitation method. Slight modifications were made to the synthesis of Titanium tetraoxide. Initially, 0.1 N of Titanium tetraoxide is dissolved in 50 ml of ethanol solution under continuous stirring for 30 min. After that, add a few drops of distilled water to form the dispersion medium. The product was placed in the ultrasonic bath for 10 min. After sonication, the solution was transferred into an autoclave at 80 °C for 24 h. Then the solution was cooled to room temperature, and it was washed and centrifuged with deionized water to remove the impurities. Then it is filtered with the Whatman No. 1 Filter paper. The synthesized TiO_2_ nanoparticles were centrifugation at 5000 rpm for 30 min and washed the pallets with distilled water three times. The pallets were oven-dried for the next 24 h at 60 °C and it is further annealed at 100 °C for 6 h. The resultant TiO_2_ NPs were collected and processed with further characterization as shown in Fig 3 [40, 41].

### Optimizing different parameters for the synthesis of TiO2 nanoparticles

The optimization of nanoparticle synthesis involves the manipulation of various parameters, including concentration, temperature, and time. Additionally, the size and morphology of the nanoparticles are influenced by factors such as concentration, temperature, and reaction time.

### Optimizing the concentration for the synthesis of TiO2 NPs

Various concentrations of the synthesized nanoparticles were optimized. The plant extract was maintained at a fixed volume while the titanium solution increased in volume proportionally. There were prepared solutions for 1:1, 1:1, 2:1, 3:1, 4:1, 5:6, 1:7, 1:8, 1:9, and 1:10. Table 1 shows that the concentration of 1:6 yielded the highest peak, with absorbance is 0.164.

**Table 1:** Optimizing of different concentrations during the synthesis of TiO_2_ NPs

Concentration	Absorbance	Time
1:1	0.012	10
1:2	0.065	20
1:3	0.064	30
1:4	0.063	40
1:5	0.062	50
1:6	0.164	60
1:7	0.062	70
1:8	0.061	80
1:9	0.060	90
1:10	0.058	100

### Time studies for the synthesis of TiO2 nanoparticles

The reaction time will affect the quality and synthesis of nanoparticles. The different periods show the synthesis of nanoparticles. Table 2 shows those 30 min were optimized and the best product of nanoparticles with absorbance is (0.369).

**Table 2:** Absorbance of different time studies for the synthesis of TiO_2_ NPs

Time (minutes)	Absorbance
10	0.277
20	0.323
30	0.369
40	0.332
50	0.275
60	0.274

### Optimized temperature for the synthesis of TiO2 NPs

Temperature plays a pivotal role in influencing nanoparticle size, shape, and overall synthesis process. In the context of green nanoparticle synthesis, moderate temperatures are preferred, whereas chemical and physical methods typically demand higher temperatures due to the involvement of numerous chemical components. Consequently, the efficient green synthesis of TiO_2_ nanoparticles necessitates careful temperature optimization. Table 3 shows that the temperature obtained at 60° C (0.146) was optimized [42].

**Table 3:** Optimized temperature for the synthesis of TiO_2_ NPs

Temperature	Absorbance	Time
20	0.023	10
40	0.069	20
60	0.146	30
80	0.028	40
100	0.026	50
120	0.025	60
140	0.024	70

### Doping with zinc nitrate

Doping is a method that modifies the chemical and physical properties of nanoparticles. It enhances the synthesis of nanoparticles. Once the optimization parameters were synthesized. TiO_2_ NPs were doping in a percent solution of zinc nitrate (0.1%, 0.2%, 0.3%, and 0.4%). Zn-doped TiO_2_ nanoparticles are to be used in different applications such as photo catalyst, antibacterial and antifungal activity [43]. Table 4 shows, that 0.4% Zn-doped TiO_2_ NPs have shown better absorbance (0.048).

**Table 4:** Different % solutions of zinc nitrate on TiO_2_ NPs

Nanoparticles	Absorbance
TiO_2_	0.021
0.1% Zn	0.024
0.2% Zn	0.031
0.3% Zn	0.036
0.4% Zn	0.048

### Characterization of TiO2 nanoparticles

The synthesized nanoparticles were characterized by using the following techniques. The characterization elaborates on the nanoparticle shape, size, and synthesis. UV/Visible spectrophotometer determines the best peak of wavelength at maximum absorbance. FTIR analysis to identify the functional group and explain the broader peaks that were forming in synthesized nanoparticles. Scanning electron microscopes investigate the surface morphology, size, and shape of nanoparticles.

1. UV/Visible Spectrophotometer (UV/Visible spectra).
2. Fourier Transform Infrared Spectrophotometer (FTIR).
3. Scanning Electron Microscopy (SEM).

### Anti-bacterial activity of Zn-doped TiO2 nanoparticles

The agar well diffusion method was used to evaluate the antibacterial activity of Zn-doped TiO_2_ nanoparticles (Rajeshkumar et al., 2014) against Gram-positive (*Staphylococcus aureus* and *Staphylococcus epidermidis*) and Gram-negative bacteria (*Salmonella* and *Escherichia coli*). Every bacterium was introduced into a medium containing nutrients [44]. Using a sterile cork borer, the culture was then evenly spread out on agar plates following the nutritional agar media’s hardening process [45]. Using a pipette, several Zn-doped TiO_2_ NP concentrations (10 mg/ml, 20 mg/ml, 25 mg/ml, and 30 mg/ml) were applied to a sterile disc. The positive control was a disc loaded with streptomycin, whereas the negative control was a disc devoid of NPs. After that, plates were incubated for 48 h at 37 °C, during which time the zone of inhibition was visible [46, 47].

### Procedure of anti-fungal activity

The anti-fungal activity of Zn-doped TiO_2_ was evaluated against different pathogenic fungi (*Aspergillus niger*,* Aspergillus flavus*,* Candida albicans*, and *Candida glabrata*) by using Sabourauds dextrose agar (SDA) method. Sabourauds dextrose agar was used for the inoculation of culture media with the suspension of fungal strains. To prepare the plates, a hole was punched with the help of a sterile cork borer into each agar, and solvent blanks were poured into the holes. Funcanzole used as a standard antibiotic was incubated in the fungal plates at 37 °C for 42 h. The inhibition of zones was measured by the diameter [48].

## Results and discussions

High-tech instruments are always being used after successful synthesis (either green, chemical, or physical methods) for the development of sustainable nanomaterials. The use of these special instruments is quite helpful for the authentic characterization compared to the standards of the prepared material showing the potential high tech. Techniques for the detailed characterization of TiO_2_ NPs synthesized by the green method can be characterized by FTIR, UV/Visible spectra, and SEM, methods for shape and surface analyses, and various results confirmed the round shape and clustered form of TiO_2_ NPs. By using SEM analysis, it is revealed that TiO_2_ NPs are spherical with small pores and normally found in the cluster that forms the bunch-type surface. Comparatively to their bulk material anatase, brookite, and rutile, they are Rhombus-like crystals, large crystals, and tetragonal in shape, respectively. In most of the cases, TiO_2_ NPs possess the above characteristics. Green TiO_2_ NPs comprised of the porous structures with comparatively large crystals showing the interesting and unique surface morphology. Owing to the porous structures, TiO_2_ Nanotubes have revolutionary applications in the environmental industry. The presence of such a unique structure is due to the presence of phytochemicals in the leaf extracts of the plant material such as terpenoids, steroids, polyphenols, flavonoids, alkaloids, antioxidants, free amino acids, and tannins. Recently synthesized the TiO_2_ NPs from the leaf extract of *Zanthoxylum Armatum*. He used the aqueous extract of the plant as well as ethanolic leaf extract for TiO_2_ NPs synthesis and compared the results with the standard parameters and noticed that the SEM of the samples that the nanoparticles prepared from the leaf extract were perfectly spherical having the nanoparticles prepared by the aqueous leaf extract. Furthermore, the author also made a comparison between TiO_2_ NPs synthesized by chemical and green methods that they also have differences in band gap energy. The morphology and elemental composition of the nanoparticles were investigated using high-resolution scanning electron microscopy (Zeiss Auriga model) operated with electron high tension at 5.0 kV for imaging.

### Optimizing different parameters for TiO2 NPs synthesis

The synthesized Zn-doped TiO_2_ nanoparticles were optimized by different parameters. The parameters affect the size, shape, and formation of NPs. Moreover, the size and shape of nanoparticles depend upon different parameters such as concentration, temperature, and time [49].

### Optimizing the different concentrations for the synthesis of TiO2 NPs

**Fig. 4:**
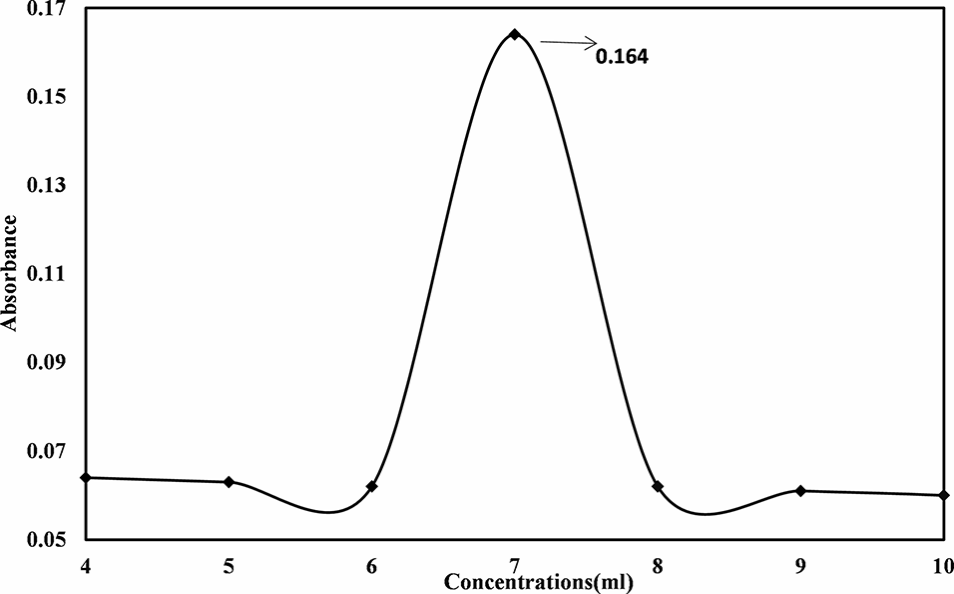
Optimizing different concentrations during the synthesis of TiO_2_ NPs

Different concentrations for the synthesis of TiO_2_ nanoparticles with *Zanthoxylum armatum* extract were prepared. The plant extract volume was kept constant while TiO_2_ was increasing by volume to ratio. Different concentrations of 1:1, 1:2,1:3,1:4,1:5,1:6,1:7,1:8,1:9 and 1:10 were prepared. In Fig 4 concentration shows the best synthesis of TiO_2_ NPs. The previous literature reported that higher concentrations produce a smaller size of the nanoparticles.

#### Time studies for the synthesis of TiO2 NPs

**Fig. 5:**
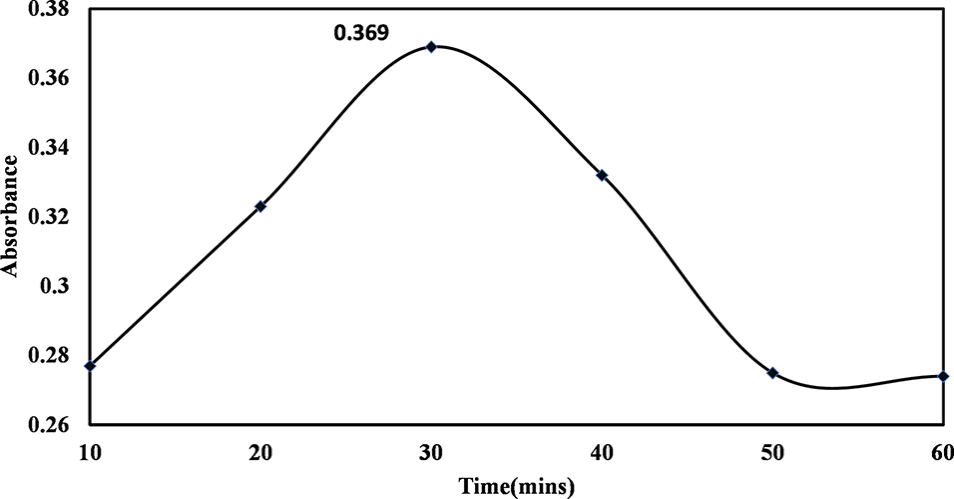
The absorbance of TiO_2_ nanoparticles at the optimum time

The synthesis of nanoparticles has also affected the reaction time. The synthesis of nanoparticles enhanced with a gradual increase in time. Fig 5, 30 min shows the optimum time for the synthesis of nanoparticles. According to previous literature, an increase in reaction time may lead to some Silver nanoparticle (AgNPs) aggregation, which could increase the size of the nanoparticles [50].

#### Optimized temperature for the synthesis of TiO2 NPs

**Fig. 6:**
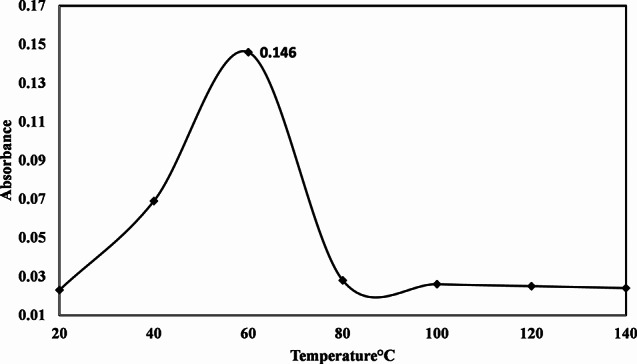
Optimized temperature for the synthesis of TiO_2_ NPs

Temperature influences the shape, size, and production of nanoparticles. The synthesis of TiO_2_ with plant extract requires a medium temperature. Because plants contain different phytochemicals that help to reduce metal ions. Fig 6 shows the 60 °C temperature determines the best synthesis of nanoparticles. Previous studies stated that at higher temperatures nanoparticles were produced in smaller sizes [51].

#### Doping with zinc nitrate

**Fig. 7:**
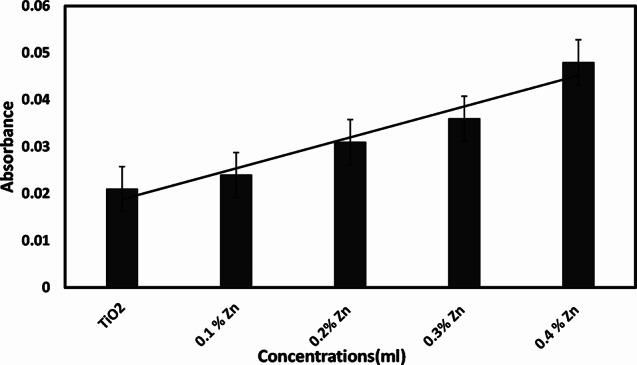
Comparison between pure TiO_2_ and Zn-doped TiO_2_

The synthesized TiO_2_ was doped with different percent solutions of Zinc nitrate. Doping enhances the nanoparticle’s properties and their applications. Fig 7 illustrates that 0.4% Zn-doped TiO_2_ determines the best result as compared to pure TiO_2_. A previous study reported that doping boosted the anti-microbial and anti-cancer activities of different metal oxide nanoparticles [52].

## Characterization

### UV/Visible spectrophotometer of TiO2 NPs

**Fig. 8:**
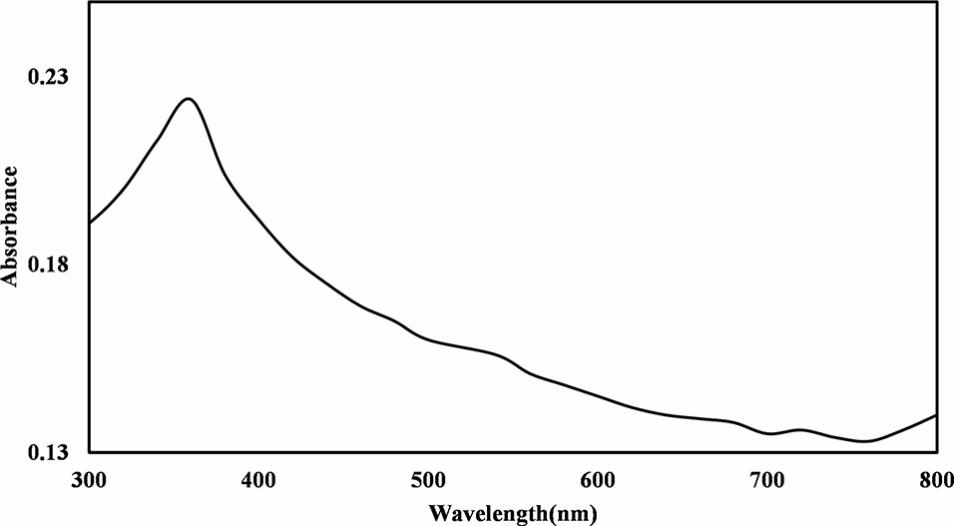
UV- vis spectra of synthesized TiO_2_ NPs

UV/Visible spectroscopy is a technique used to analyze and quantify the absorption of ultraviolet (UV) and visible light by chemical compounds. The interaction between light and matter occurs due to the energy level of electrons within the atoms or molecules of the sample. UV/Visible spectroscopy’s basic idea is based on how various chemical substances absorb UV or visible light to create distinct spectra. When the electron goes to the excitation state, the matter absorbs ultraviolet radiation due to this it goes from the ground state to the energized state. The different metal oxide nanoparticles work on different wavelengths. The synthesized TiO_2_ NPs were examined by using a UV/Visible spectrophotometer in the region of 200 nm to 800 nm. The foundation of this technique lies in the principles of adsorption spectroscopy. According to literature reported that TiO_2_ peaks lie between 380 nm and 400 nm. Fig 8 demonstrated that a strong peak at 360 nm with (0.224) absorbance confirmed the synthesis of TiO_2_ NPs using *Zanthoxylum armatum* leaf extract.

### Fourier transform infrared (FTIR) spectroscopy

**Fig. 9:**
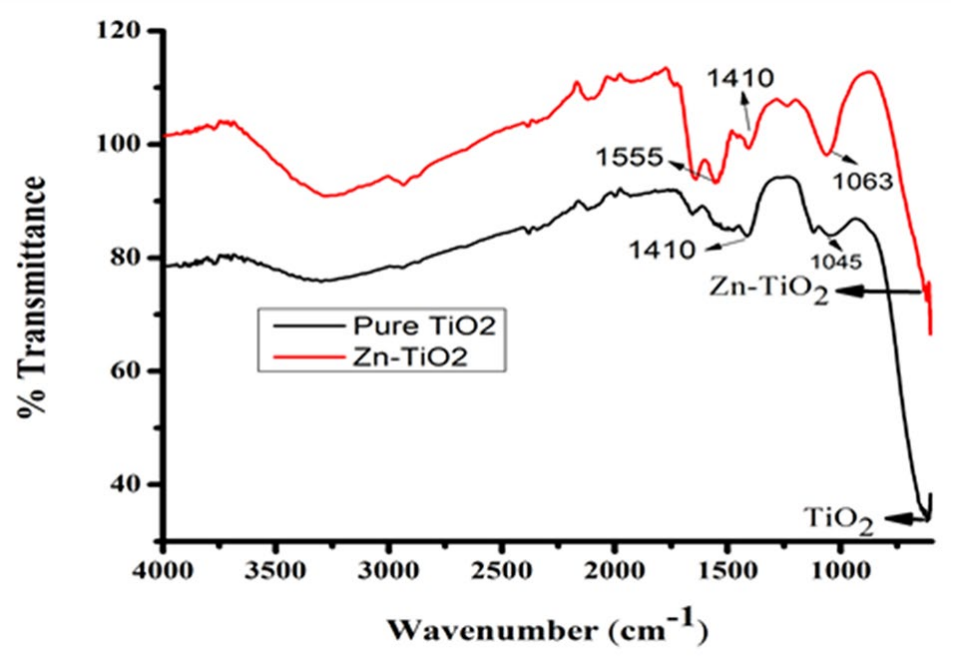
FTIR analysis of pure TiO_2_ and Zn-TiO_2_ NPs

The functional group and chemical compound present in the prepared TiO_2_ NPs were identified using the FTIR spectrum as shown in Fig 9. The broadband at 600 cm^− 1^ belongs to TiO_2_ and correlates to the O-H stretching vibration. The bands are 1410 cm^− 1^, 1045 cm^− 1^, 1410 cm^− 1^_,_ 1063 cm^− 1^, and 1555 cm^− 1^ corresponding to aromatic stretching vibration. The strong band at 900 cm^− 1^ reveals the formation of Ti-O-Ti bending vibrations, respectively. The metal oxide bonds like Ti-O-Ti confirms the existence of TiO_2_ in the prepared TiO_2_ NPs. The FT-IR spectrum confirms the presence of TiO_2_, indicated by the two peaks at 675 cm^− 1^ and 1610 cm^− 1^, corresponding to the bending of Ti-O and the stretching of Ti-OH, respectively. Nanocrystalline TiO_2_ was prepared by sol-gel technique. Direct, and indirect band gap energies and particle size were evaluated. Pure TiO_2_ nanoparticles were synthesized by sol-gel technique at room temperature with appropriate reactants. The synthesis of anatase phase TiO_2_ nanoparticles was achieved by tetra is-propyl as a common starting material and the product was annealed. The FTIR spectrum of ZnTiO_2_ nanoparticles the peaks located at 565 and 960 cm^− 1^ are related to metal oxide vibrations of Zn-O bonds, and the peaks obtained at 1018 cm^− 1^ are related to the Zn-O stretch bond. The spectrum of ZnONP is characterized by a strong absorption in a UV band extending considerably into the UV range, with a local absorption peak at 362 nm, whereas absorption is characterized by a relatively narrower spectral band. The presence of the Ti-O-Ti bond is due to the strong interaction (capped) of biomolecules with TiO_2_ NPs which results in the presence of alkaloids. These phytochemicals are responsible for reducing the bulk of titanium dioxide to stable TiO_2_ in green synthesis. The hydroxyl groups present in TiO_2_ NPs, enhance the.

photocatalytic performance. Several kinds of literature have documented that dopants with higher atomic numbers tend to cause a shift of FTIR bands towards lower wavenumbers [53].

### Scanning electron microscope (SEM)

The SEM images of prepared TiO_2_ NPs. The SEM image of bio-mediated TiO_2_ nanoparticles is a spherical-shaped structure and the chemical synthesis TiO_2_ nanoparticles has sphere-like surface morphology. This method utilizes electrons to generate a micro-image observable through a microscope. Positioned at the uppermost micro-column are the electron source and lens. As a result, primary and secondary electrons are released when the electrons are stimulated by the electron beam. The SEM image of bio-mediated TiO_2_ nanoparticles is a spherical-shaped structure and the chemical synthesis of TiO_2_ nanoparticles has sphere-like surface morphology. It is a type of electron microscopy that uses a focused beam of electrons to scan the surface of a specimen and generate images at a much greater resolution compared to optical microscopy. The resolution of SEM instruments can range from < 1 nm up to several nanometers. The morphology of the sample is shown in Fig. 3. The particle size of the sample is about 15 nm. The sizes of the particles and the surfaces of the samples did not differ significantly so the SEM images of the pure TiO_2_ and the Zn-TiO_2_ are shown. When the amount of Zn doping was increased, the surface of the sample was agglomerated. All of the samples used the same amount of ammonium at a controlled pH in the reaction. Therefore, when the amount of Zn doping was increased, the pH decreased because the ammonium was diluted due to the additional water in the zinc solution. This indicates that the repulsion of TiO_2_ units decreased, resulting in a large, agglomerated particle network. However, the network of the particle affects not only the pH but also the amount of water. By increasing the Zn doping, the total amount of water in the reaction increased, so the space needed for TiO_2_. This method utilizes electrons to generate a micro-image observable through a microscope. Positioned at the uppermost micro-column are the electron source and lens. As a result, primary and secondary electrons are released when the electrons are stimulated by the electron beam. According to earlier research, investigate that the SEM of TiO_2_ NPs showed that were generally found in the cluster and spherical form with few pores. Zn-doped TiO_2_ exhibits in cluster and spherical shape. There was a minor aggregation. In general, the decrease in particle size is inversely proportional to the surface volume of the material. Therefore, the lower particle size material quickly penetrates the toxic elements and the bacterial surface, leading to the decomposition process.

**Fig. 10:**
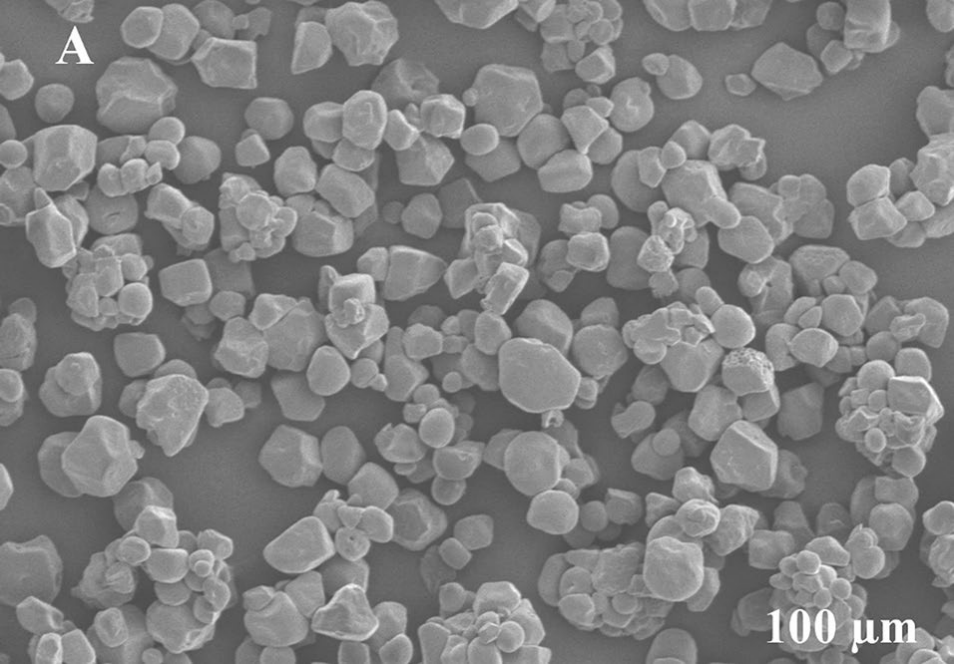
(**A**) SEM of synthesized Zn-doped TiO_2_ NPs

According to earlier research, investigate that the SEM of TiO_2_ NPs showed that were generally found in the cluster and spherical form with few pores. Fig 10 revealed that Zn-doped TiO_2_ exhibits in cluster and spherical shape. There was a minor aggregation. In general, the decrease in particle size is inversely proportional to the surface volume of the material. Therefore, the lower particle size material quickly penetrates the toxic elements and the bacterial surface, leading the decomposition process [54].

### Anti-bacterial activity of TiO2 and Zn-doped TiO2 NPs

**Fig. 11:**
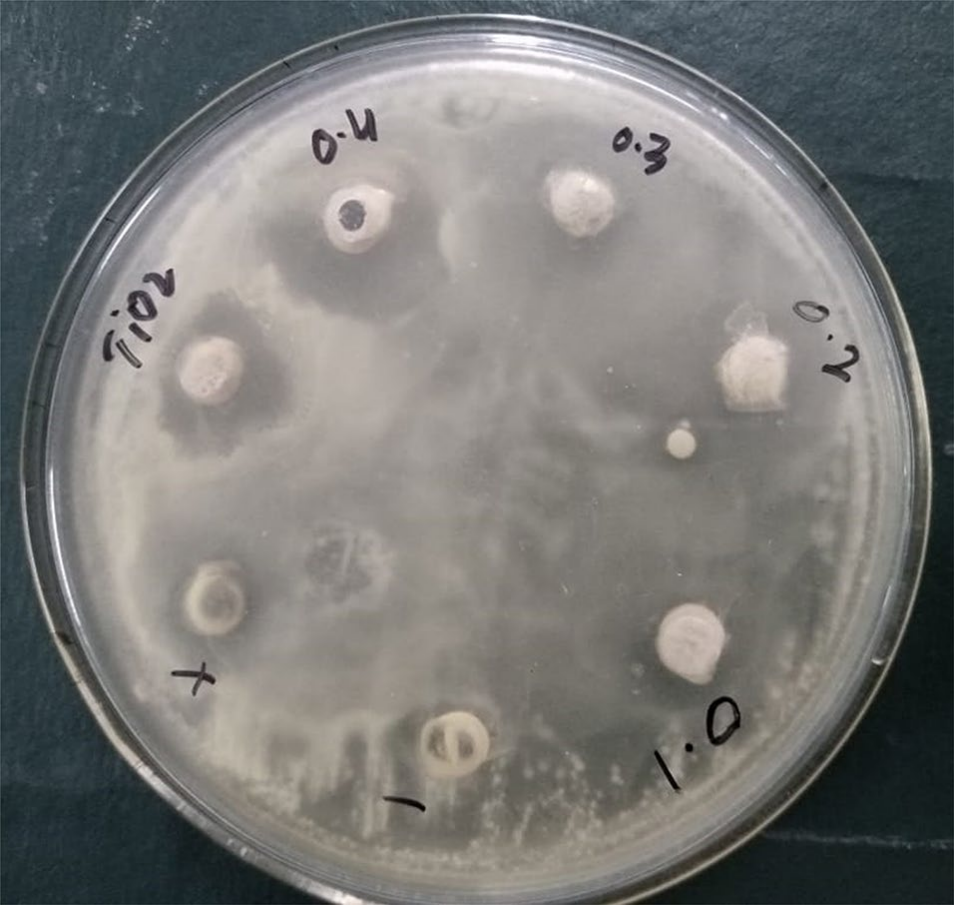
Inhibition images of antibacterial activities

**Fig. 12:**
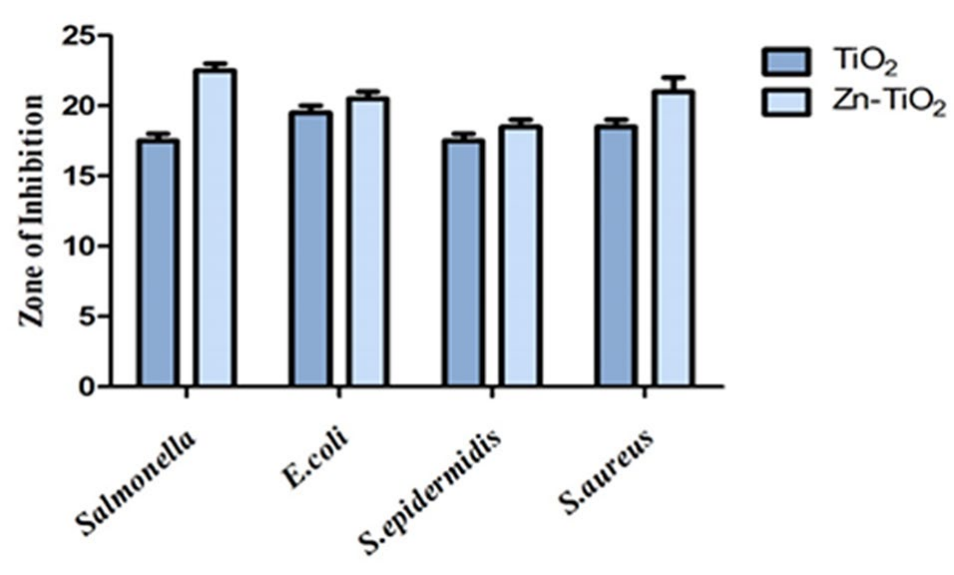
Anti-bacterial activity of synthesized Zn-doped TiO_2_ NPs

The Zn-doped TiO_2_ NPs antibacterial activity was produced by employing the *Zanthoxylum armatum* plant was evaluated by selecting two gram-ve bacteria (*Salmonella* and *Escherichia coli*) and two gram-ve bacteria (*Staphylococcus epidermidis* and *Staphylococcus aureus*). The investigation showed that Zn-doped TiO_2_ nanoparticles caused the zone of inhibition to expand as shown in Fig 11. Gram-positive bacteria have a thicker peptidoglycan layer on their cell walls, whereas Gram-negative bacteria have a thin one. ZOI was measured on a millimeter scale [55]. Compared to Gram-positive bacteria, gram-negative bacteria have a thinner and less robust cell wall, which suggests that they are less capable of preventing the entry of harmful species into the cytoplasm. The results indicate that the Zn-doped TiO_2_ nanoparticles form an enhanced zone of inhibition, as shown in Table 5. Gram-negative bacteria showed the highest zone of inhibition (*Salmonella* and *Escherichia coli*) in Zn-TiO_2,_ and the gramme + ve bacteria showed the smallest zone of inhibition (S. *epidermidis* and *S. aureus*) in pure TiO_2_ as shown in Fig 12. When compared to gram-positive bacteria, gram-negative bacteria are much more powerful because of differences in the synthesis process, bacterial susceptibility, nanoparticle morphology, phase formations, particle size, shape, and variability in the zone of inhibition’s diameter [56]. The strong antibacterial activity can suppress positive and negative bacteria growth. Previous research indicates that Zn-doped TiO_2_ NPs possess potential anti-bacterial activity [57].

**Table 5:** Zone of inhibition

Nanoparticles	*Salmonella*	*E. coli*	*S. epidermidis*	*S. aureus*
TiO_2_	17	19	17	18
Zn-TiO_2_	23	21	19	22

### Anti-fungal activity of TiO2 and Zn-doped TiO2 NPs

**Fig. 13:**
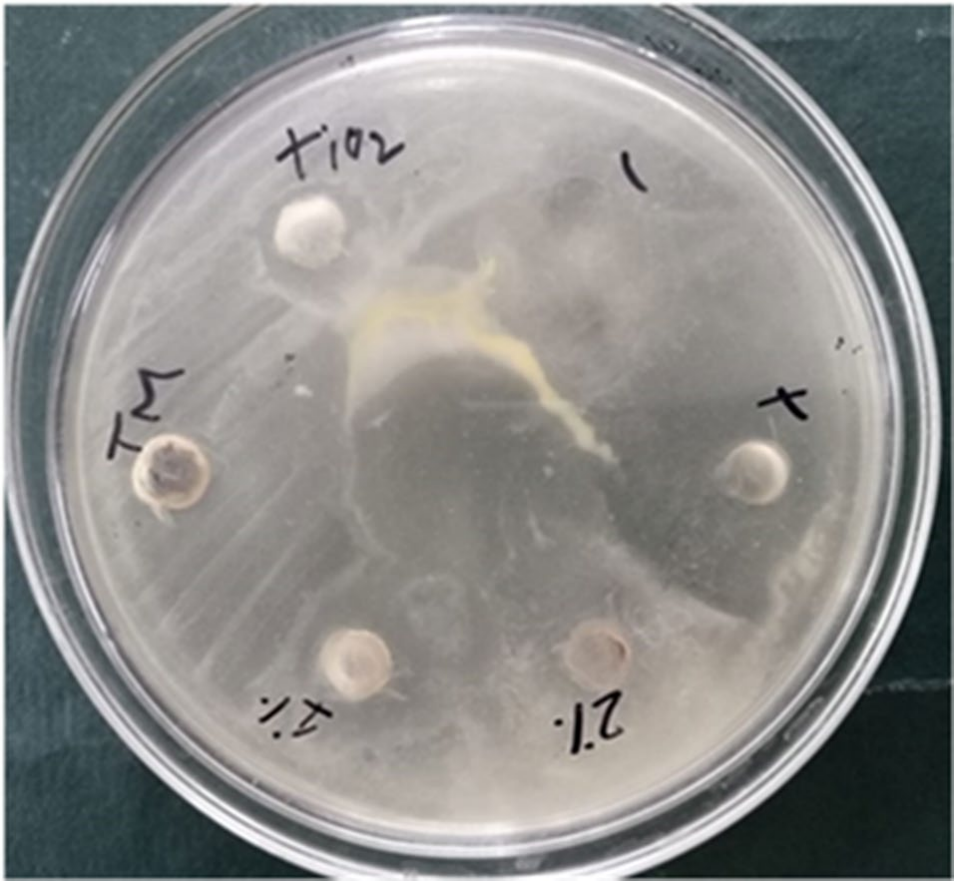
Inhibition images of anti-fungal activities

Multiple factors have contributed to the emergence of microbial resistance to antibiotics, which has increased the current demand for more advanced antimicrobial medicines. The antifungal activity of biosynthesis of Zn-doped TiO_2_ was estimated by the diameter of the zone of inhibition in millimeters (mm). Zn-doped TiO_2_ exhibited powerful antifungal activity towards *Aspergillus flavus*,* Aspergillus niger*,* Candida glabrata*, and *Candida albicans*. Table 6 result revealed that the green synthesis of Zn-doped TiO_2_ NPs shows great antifungal activity against multicellular (*Candida glabrata* and *Candida albicans*) and unicellular fungi (*Aspergillus flavus* and *Aspergillus niger*). In Fig 13, the results confirmed that Zn-doped TiO_2_ nanoparticles have good antifungal activity as compared to Pure TiO_2_ because of their doping [58].

**Fig. 14:**
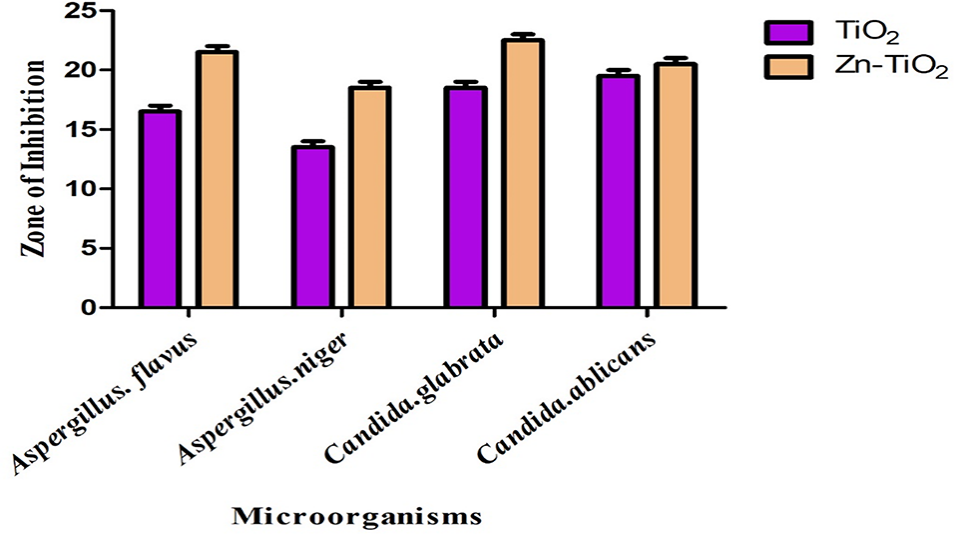
Anti-fungal activity of synthesized Zn-doped TiO_2_ NPs

Doping enhances the photocatalytic activity of Zn-doped TiO_2_ NPs as shown in Fig 14. This study revealed that the properties of TiO_2_ were significantly enhanced by the doping of the meal ions and enhanced antifungal activity [59]. Prior studies investigated that the expansion of the zone of inhibition depends upon both the nanoparticle concentrations and fungal growth. Additionally, this phenomenon was influenced by the concentrations of fungal spores [60].

**Table 6:** Zone of inhibition of different fungus

Microorganisms	TiO_2_ NPs	Zn-TiO_2_ NPs
*Aspergillus. Flavus*	17	22
*Aspergillus. Niger*	14	19
*Candida. Glabrata*	19	23
*Candida. Ablicans*	20	21

## Conclusion

The creation of Zn-doped TiO_2_ nanoparticles is the focus of this work on *Zanthoxylum armatum* DC. In this present work, TiO_2_ NPs are successfully synthesized by green synthesis and hydrothermal methods (chemical method). Color changes confirmed the reduction of bulk Titanium to nanoparticles. The photodegradation under UV-visible irradiation results in the degradation. Bio-mediated TiO_2_ shows a maximum degradation efficiency of irradiation. SEM image reveals a uniform spherical shape surface morphology. The antibacterial activity of TiO_2_ NPs was visualized by the agar diffusion method. The antibacterial activity of TiO_2_ NPs was tested against bacterial pathogens such as Staphylococcus aureus (gram-positive bacteria) Escherichia coli and Klebsiella pneumonia (gram-negative bacteria). The bio-mediated TiO_2_ NPs exhibit a good potent antibacterial activity. The suggested results have inferred the property of TiO_2_ nanoparticles is suited for biomedical applications. Furthermore, the properties of TiO_2_ nanoparticles were significantly enhanced by the doping of metal oxide therefore, the doped showed better results as compared to the un-doped. The doped metal oxide nanoparticles. Zn-doped TiO_2_ nanoparticles have strong anti-microbial activity as compared to Pure TiO_2_. Zn-doped TiO_2_ was used in many anti-microbial applications.

## Data Availability

All the data is available within the manuscript.

## Abbreviations

TiO_2_: Titanium dioxide
NPs: Nanoparticles
ROS: Reactive Oxygen Species
UV/Visible spectra: UV/Visible Spectrophotometer
FTIR: Fourier Transform Infrared Spectrophotometer
SEM: Scanning Electron Microscopy
SDA: Sabourauds dextrose agar
AgNPs: Silver nanoparticles
